# 2019 update of the WSES guidelines for management of *Clostridioides* (*Clostridium*) *difficile* infection in surgical patients

**DOI:** 10.1186/s13017-019-0228-3

**Published:** 2019-02-28

**Authors:** Massimo Sartelli, Stefano Di Bella, Lynne V. McFarland, Sahil Khanna, Luis Furuya-Kanamori, Nadir Abuzeid, Fikri M. Abu-Zidan, Luca Ansaloni, Goran Augustin, Miklosh Bala, Offir Ben-Ishay, Walter L. Biffl, Stephen M. Brecher, Adrián Camacho-Ortiz, Miguel A. Caínzos, Shirley Chan, Jill R. Cherry-Bukowiec, Jesse Clanton, Federico Coccolini, Maria E. Cocuz, Raul Coimbra, Francesco Cortese, Yunfeng Cui, Jacek Czepiel, Zaza Demetrashvili, Isidoro Di Carlo, Salomone Di Saverio, Irina M. Dumitru, Christian Eckmann, Edward H. Eiland, Joseph D. Forrester, Gustavo P. Fraga, Jean L. Frossard, Donald E. Fry, Rita Galeiras, Wagih Ghnnam, Carlos A. Gomes, Ewen A. Griffiths, Xavier Guirao, Mohamed H. Ahmed, Torsten Herzog, Jae Il Kim, Tariq Iqbal, Arda Isik, Kamal M. F. Itani, Francesco M. Labricciosa, Yeong Y. Lee, Paul Juang, Aleksandar Karamarkovic, Peter K. Kim, Yoram Kluger, Ari Leppaniemi, Varut Lohsiriwat, Gustavo M. Machain, Sanjay Marwah, John E. Mazuski, Gokhan Metan, Ernest E. Moore, Frederick A. Moore, Carlos A. Ordoñez, Leonardo Pagani, Nicola Petrosillo, Francisco Portela, Kemal Rasa, Miran Rems, Boris E. Sakakushev, Helmut Segovia-Lohse, Gabriele Sganga, Vishal G. Shelat, Patrizia Spigaglia, Pierre Tattevin, Cristian Tranà, Libor Urbánek, Jan Ulrych, Pierluigi Viale, Gian L. Baiocchi, Fausto Catena

**Affiliations:** 1 Department of Surgery, Macerata Hospital, Via Santa Lucia 2, 62100 Macerata, Italy; 2 Infectious Diseases Department, Trieste University Hospital, Trieste, Italy; 3 Medicinal Chemistry, School of Pharmacy, University of Washington, Seattle, WA USA; 4 Division of Gastroenterology and Hepatology, Department of Medicine, Mayo Clinic, Rochester, MN USA; 5 Research School of Population Health, Australian National University, Acton, ACT Australia; 6 Department of Microbiology, Faculty of Medical Laboratory Sciences, Omdurman Islamic University, Khartoum, Sudan; 7 Department of Surgery, College of Medicine and Health Sciences, UAE University, Al-Ain, United Arab Emirates; 8 Department of General Surgery, Bufalini Hospital, Cesena, Italy; 9 Department of Surgery, University Hospital Centre Zagreb and School of Medicine, University of Zagreb, Zagreb, Croatia; 10 Trauma and Acute Care Surgery Unit, Hadassah Hebrew University Medical Center, Jerusalem, Israel; 11 Department of General Surgery, Rambam Health Care Campus, Haifa, Israel; 12 Trauma and Acute Care Surgery, Scripps Memorial Hospital La Jolla, La Jolla, CA USA; 13 Pathology and Laboratory Medicine, VA Boston Healthcare System, West Roxbury MA and BU School of Medicine, Boston, MA USA; 14 Department of Internal Medicine, University Hospital, Dr. José E. González, Monterrey, Mexico; 15 Department of Surgery, University of Santiago de Compostela, A Coruña, Spain; 16 Department of General Surgery, Medway Maritime Hospital, Gillingham, Kent UK; 17 Department of Surgery, Division of Acute Care Surgery, University of Michigan, Ann Arbor, MI USA; 18 Department of Surgery, West Virginia University Charleston Division, Charleston, WV USA; 19 Faculty of Medicine, Transilvania University, Infectious Diseases Hospital, Brasov, Romania; 20 Riverside University Health System Medical Center and Loma Linda University School of Medicine, Moreno Valley, CA USA; 21 Emergency Surgery Unit, San Filippo Neri’s Hospital, Rome, Italy; 22 Department of Surgery, Tianjin Nankai Hospital, Nankai Clinical School of Medicine, Tianjin Medical University, Tianjin, China; 23 Department of Infectious Diseases, Jagiellonian University, Medical College, Kraków, Poland; 24 Department of Surgery, Tbilisi State Medical University, Kipshidze Central University Hospital, Tbilisi, Georgia; 25 Department of Surgical Sciences, Cannizzaro Hospital, University of Catania, Catania, Italy; 26 Department of Surgery, Addenbrooke’s Hospital, Cambridge University Hospitals NHS Foundation Trust, Cambridge, UK; 27 Clinical Infectious Diseases Hospital, Ovidius University, Constanta, Romania; 28 Department of General, Visceral and Thoracic Surgery, Klinikum Peine, Hospital of Medical University Hannover, Peine, Germany; 29 Vital Care, Inc, Meridian, MS USA; 30 Department of Surgery, Stanford University, Stanford, CA USA; 31 Division of Trauma Surgery, Hospital de Clinicas, School of Medical Sciences, University of Campinas, Campinas, Brazil; 32 Service of Gastroenterology and Hepatology, Geneva University Hospital, Genève, Switzerland; 33 Department of Surgery, Northwestern University Feinberg School of Medicine, Chicago, IL USA; 34 University of New Mexico School of Medicine, Albuquerque, NM USA; 35 Critical Care Unit, Instituto de Investigación Biomédica de A Coruña (INIBIC), Complexo Hospitalario Universitario de A Coruña (CHUAC), Sergas, Universidade da Coruña (UDC), A Coruña, Spain; 36 Department of Surgery Mansoura, Faculty of Medicine, Mansoura University, Mansoura, Egypt; 37 Surgery Department, Hospital Universitario (HU) Terezinha de Jesus da Faculdade de Ciencias Medicas e da Saude de Juiz de Fora (SUPREMA), Hospital Universitario (HU) Universidade Federal de Juiz de Fora (UFJF), Juiz de Fora, Brazil; 38 Department of Surgery, Queen Elizabeth Hospital, Birmingham, UK; 39 Unit of Endocrine, Head, and Neck Surgery and Unit of Surgical Infections Support, Department of General Surgery, Parc Taulí, Hospital Universitari, Sabadell, Spain; 40 Department of Medicine, Milton Keynes University Hospital NHS Foundation Trust, Milton Keynes, Buckinghamshire UK; 41 Department of Surgery, St. Josef Hospital, Ruhr University Bochum, Bochum, Germany; 42 Department of Surgery, Ilsan Paik Hospital, Inje University College of Medicine, Goyang, Republic of Korea; 43 Department of Gastroenterology, Queen Elizabeth Hospital, Birmingham, UK; 44 General Surgery Department, Magee Womens Hospital, UPMC, Pittsburgh, USA; 45 Department of Surgery, VA Boston Health Care System, Boston University and Harvard Medical School, Boston, MA USA; 46 Global Alliance for Infections in Surgery, Porto, Portugal; 47 School of Medical Sciences, University Sains Malaysia, Kota Bharu, Kelantan Malaysia; 48 Department of Pharmacy Practice, St Louis College of Pharmacy, St Louis, MO USA; 49 Faculty of Mediine University of Belgrade Clinic for Surgery “Nikola Spasic”, University Clinical Center “Zvezdara” Belgrade, Belgrade, Serbia; 50 Department of Surgery, Jacobi Medical Center, Albert Einstein College of Medicine, Bronx, NY USA; 51 Abdominal Center, Helsinki University Hospital Meilahti, Helsinki, Finland; 52 Department of Surgery, Faculty of Medicine Siriraj Hospital, Mahidol University, Bangkok, Thailand; 53 Department of Surgery, Universidad Nacional de Asuncion, Asuncion, Paraguay; 54 Department of Surgery, Post-Graduate Institute of Medical Sciences, Rohtak, India; 55 Department of Surgery, Washington University School of Medicine, Saint Louis, USA; 56 Department of Infectious Diseases and Clinical Microbiology, Hacettepe University Faculty of Medicine, Ankara, Turkey; 57 Department of Surgery, University of Colorado, Denver Health Medical Center, Denver, CO USA; 58 Department of Surgery, University of Florida, Gainesville, FL USA; 59 Department of Surgery, Fundación Valle del Lili, Hospital Universitario del Valle, Universidad del Valle, Cali, Colombia; 60 Infectious Diseases Unit, Bolzano Central Hospital, Bolzano, Italy; 61 National Institute for Infectious Diseases - INMI - Lazzaro Spallanzani IRCCS, Rome, Italy; 62 Gastroenterology Department, Centro Hospitalar e Universitário de Coimbra, Coimbra, Portugal; 63 Department of Surgery, Anadolu Medical Center, Kocaali, Turkey; 64 Department of Abdominal and General Surgery, General Hospital Jesenice, Jesenice, Slovenia; 65 Department of Surgery, Medical University of Plovdiv, Plovdiv, Bulgaria; 66 Division of Emergency Surgery, Department of Surgery, Fondazione Policlinico Universitario A. Gemelli IRCCS, Rome, Italy; 67 Department of Surgery, Tan Tock Seng Hospital, Singapore, Singapore; 68 Department of Infectious Diseases, Istituto Superiore di Sanità, Rome, Italy; 69 Infectious Diseases and Intensive Care Unit, Pontchaillou University Hospital, Rennes, France; 70 First Department of Surgery, Faculty of Medicine, Masaryk University Brno and University Hospital of St. Ann Brno, Brno, Czech Republic; 71 First Department of Surgery, First Faculty of Medicine, Charles University in Prague and General University Hospital in Prague, Prague, Czech Republic; 72 Clinic of Infectious Diseases, St Orsola-Malpighi University Hospital, Bologna, Italy; 73 Department of Clinical and Experimental Sciences, University of Brescia, Brescia, Italy; 74 Emergency Surgery Department, Maggiore Parma Hospital, Parma, Italy

**Keywords:** Clostridioides difficile infection, *Clostridium difficile* infection, Pseudomembranous colitis, Antimicrobial treatment, Fecal microbiota transplantation, Infection control, Antimicrobial stewardship

## Abstract

In the last three decades, *Clostridium difficile* infection (CDI) has increased in incidence and severity in many countries worldwide. The increase in CDI incidence has been particularly apparent among surgical patients. Therefore, prevention of CDI and optimization of management in the surgical patient are paramount. An international multidisciplinary panel of experts from the World Society of Emergency Surgery (WSES) updated its guidelines for management of CDI in surgical patients according to the most recent available literature. The update includes recent changes introduced in the management of this infection.

## Introduction

In the last three decades, the dramatic worldwide increase in incidence and severity of *Clostridium difficile* infection (CDI) [1] has made CDI a global public health challenge [2–14]. Surgery is a known risk factor for development of CDI yet surgery is also a treatment option in severe cases of CDI [15–18]. The World Society of Emergency Surgery (WSES) guidelines for management of CDI in surgical patients were published in 2015 [19]. In 2019, the guidelines have been revised and updated. A multidisciplinary expert panel worldwide prepared the manuscript following an in-depth review of the most recent current literature using MEDLINE, EMBASE, and Cochrane Database and aimed to provide an insight into these complex issues. The expert panel met via email to prepare, discuss, and revise the paper. The manuscript was successively reviewed by all members and ultimately re-formulated as the present manuscript.

These guidelines outline clinical recommendations based on the Grading of Recommendations Assessment, Development, and Evaluation (GRADE) hierarchy criteria from Guyatt et al. [20, 21] (Table 1).

**Table 1:** Grading of recommendations from Guyatt and colleagues [20, 21]

Grade of recommendation	Clarity of risk/benefit	Quality of supporting evidence	Implications
1A			
Strong recommendation, high-quality evidence	Benefits clearly outweigh risk and burdens, or vice versa	RCTs without important limitations or overwhelming evidence from observational studies	Strong recommendation, applies to most patients in most circumstances without reservation
1B			
Strong recommendation, moderate-quality evidence	Benefits clearly outweigh risk and burdens, or vice versa	RCTs with important limitations (inconsistent results, methodological flaws, indirect analyses or imprecise conclusions) or exceptionally strong evidence from observational studies	Strong recommendation, applies to most patients in most circumstances without reservation
1C			
Strong recommendation, low-quality or very low-quality evidence	Benefits clearly outweigh risk and burdens, or vice versa	Observational studies or case series	Strong recommendation but subject to change when higher quality evidence becomes available
2A			
Weak recommendation, high-quality evidence	Benefits closely balanced with risks and burden	RCTs without important limitations or overwhelming evidence from observational studies	Weak recommendation, best action may differ depending on the patient, treatment circumstances, or social values
2B			
Weak recommendation, moderate-quality evidence	Benefits closely balanced with risks and burden	RCTs with important limitations (inconsistent results, methodological flaws, indirect or imprecise) or exceptionally strong evidence from observational studies	Weak recommendation, best action may differ depending on the patient, treatment circumstances, or social values
2C			
Weak recommendation, low-quality or very low-quality evidence	Uncertainty in the estimates of benefits, risks, and burden; benefits, risk, and burden may be closely balanced	Observational studies or case series	Very weak recommendation; alternative treatments may be equally reasonable and merit consideration

C*lostridioides difficile* (formerly *Clostridium difficile*) is an anaerobic, spore-forming, Gram-positive bacillus, which may be part of the normal intestinal microbiota in healthy babies [22–25]. The organism is spread via the oral-fecal route and in hospitalized patients may be acquired through the ingestion of spores from other patients, healthcare personnel’s hands, or from environmental surfaces [26, 27]. *C*. *difficile* is the main pathogen associated with nosocomial infections and is the most common cause of diarrhea in hospitalized patients [28]. CDI can present as a spectrum of symptoms ranging from an asymptomatic carriage to fulminant disease with toxic megacolon. The basis for this range of clinical manifestations is not fully understood but is likely related to host and pathogen interactions.

The rapid evolution of antibiotic resistance in *C*. *difficile* and the consequent effects on prevention and treatment of CDIs are a matter of concern for public health. Multi-drug resistant (MDR) *C*. *difficile* strains are increasing (about 60% of the epidemic strains circulating in hospital settings show resistance to three or more antibiotics) [29].

## Pathogenesis

*C*. *difficile* spores survive the acidic environment of the stomach and germinate in the intestine [30], which act as a reservoir for *C*. *difficile* and can facilitate spread among patients, as well as contribute to the high recurrence rates observed in CDI. The primary toxins produced by this bacterium are toxins A and B [31]. Toxins A and B act as glucosyltransferases, promoting the activation of Rho GTPases leading to disorganization of the cytoskeleton of the colonocyte, and eventual cell death [32]. Since CDI is a toxin-mediated infection, non-toxigenic *C*. *difficile* strains are non-pathogenic. The respective roles and importance of toxins A and B have been debated. Toxin A was thought to be the major virulence factor for many years [33–35]. It is now established that both toxins A and B are important for inducing colonocyte death and colitis, and there is increasing evidence pointing toward their role in CDI extra-intestinal effects [36]. In addition to toxins A and B, some strains produce a third toxin known as binary toxin [37–41]. Binary toxin has an ADP-ribosyltransferase function, which also leads to actin depolymerization [42, 43]. However, its pathogenetic role is still debated [44, 45].

Asymptomatic *C*. *difficile* colonization occurs when *C*. *difficile* is detected in the absence of symptoms of infection. Asymptomatic colonized individuals with no clinical signs of CDI can still act as an infection reservoir and transmit *C*. *difficile* to others [46, 47]. Asymptomatic colonization with *C*. *difficile* may be a crucial factor in the progression to CDI, as carriers of toxigenic strains may be at a higher risk for the development of an infection compared to non-colonized patients [48]. Other data suggests that carriage of non-toxigenic *C*. *difficile* may be protective against toxigenic ribotypes [49]. Estimates of prevalence of asymptomatic *C*. *difficile* colonization vary considerably between different patient groups. Among healthy adults with no prior risk factors for CDI, asymptomatic colonization prevalence varied between 0 and 15% [50–56].

## Risk factors

Risk factors for CDI may be divided into three general categories: host factors (immune status, comorbidities), exposure to *C*. *difficile* spores (hospitalizations, community sources, long-term care facilities), and factors that disrupt normal colonic microbiome (antibiotics, other medications, surgery) [57].

### Patient factors

Risk factors identified to date include age > 65 years, comorbidity or underlying conditions, inflammatory bowel diseases, immunodeficiency (including human immunodeficiency virus infection), malnutrition, obesity, female sex, and low serum albumin level [3, 58]. Patients with comorbidities may have distinct characteristics of their CDI, for example, in type 2 diabetes mellitus, patients with CDI were younger, and sepsis and proton pump inhibitors (PPIs) were important causes, but fever was not a dominant feature [59].

The effects of prior appendectomy on the development of *C*. *difficile* colitis have been debated [60]. A review by Seretis et al. [61] of five studies conducted retrospectively and published in 2014 reported that an in situ appendix did not impact on the development of CDI. In the retrospective analysis by Clanton et al. [62] on 55 patients who underwent colectomy for CDI between 2001 and 2011, a prior appendectomy was noted in 24 of 55 patients (44%, 99% CI 0.280–0.606). In another retrospective study of 507 patients [63], 13 of 119 patients (10.9%) with a previous appendectomy required colectomy compared to 20 of 388 patients (5.2%) with an intact appendix developing fulminant infection and requiring colectomy and increased disease severity, indicated by increased rates of colectomy, occurred in the group with a history of appendectomy (*p* = 0.03). A sub-group analysis of a large population-based study published in 2013 [64] showed that appendectomy was not associated with adverse outcomes in CDI. Patients with appendectomy before CDI showed no differences in risk factors, treatment, or outcomes including treatment failure, development of severe or severe-complicated CDI, or recurrence rates as compared with patients without appendectomy. Larger prospective studies are needed to assess the impact of prior appendectomy on the development and severity of CDI.

### Exposure to *Clostridium difficile* spores

Factors that increase risk of exposure to *C*. *difficile* spores, such as increased duration of hospital stay, increase the risk of CDI. A length of stay > 2 weeks has been shown to be a risk factor for CDI [65]. Hospitals with well-implemented infection prevention and control measures are at lower risk of nosocomial CDI [66].

### Normal flora disruption

The indigenous gut microbiota is a complex community of microorganisms that populates the gastrointestinal tract in a healthy person. This micro-ecosystem plays a crucial role in protecting the intestines by providing resistance to colonization and infection by pathogenic organisms [67]. Gut microbiota has also immeasurable effects on homeostasis of the host [68]. Under normal conditions, the human gut microbiota may impede pathogen colonization through general mechanisms such as direct inhibition through bacteriocins, nutrient depletion (consuming growth-limiting nutrients), or stimulation of host immune defenses [57], though the exact mechanism by which the microbiota protects against CDI is unknown [69]. Disruption of the normal balance of colonic microbiota as a consequence of antibiotic use or other stressors is, however, of major importance [70].

#### Antibiotic exposure

Disruption of the normal gut flora allows *C*. *difficile* to proliferate and produce toxins. In 1974, Tedesco et al. published a prospective study of clindamycin-associated colitis, which had become endemic in many hospitals [71]. In 200 consecutive patients, administration of clindamycin resulted in diarrhea in 21% and the incidence of endoscopy-diagnosed pseudomembranous colitis was 10%. The study led to a search for an infectious cause of colitis, and it identified *C. difficile* as the main causative agent [72].

The risk of CDI is increased up to sixfold during antibiotic therapy and in the subsequent month afterwards [73]. Although nearly all antibiotics have been associated with CDI, clindamycin, third-generation cephalosporins, penicillins, and fluoroquinolones have traditionally been considered to pose the greatest risk [74–80]. An association between CDI and antimicrobial treatment > 10 days has also been demonstrated [81, 82]. Antibiotics which have been less commonly associated with CDI include macrolides, sulfonamides, and tetracyclines [83]. Even very limited exposure, such as single-dose surgical antibiotic prophylaxis, can increase patients risk for both *C*. *difficile* colonization or infection [84–86].

#### Other medications

Exposure to gastric acid-suppressive medications, such as histamine-2 blockers and PPIs, may be a potential risk factor for development of CDI. Several studies have suggested the association between use of stomach acid-suppressive medications, primarily PPIs, and CDI [87, 88]. In 2012, a systematic review of incident and recurrent CDI in PPI users was published [89]. Forty-two observational studies (30 case-control, 12 cohort) totaling 313,000 participants were evaluated. Despite the substantial statistical and clinical heterogeneity, the findings indicated a probable association between PPI use and incident and recurrent CDI. This risk was further increased by concomitant use of antibiotics and PPI. Other studies suggested that this association may be the result of confounding with the underlying severity of illness and duration of hospital stay [90]. Another meta-analysis about a plausible link between CDI and PPIs was recently published [91]. Pooled analysis of 50 studies showed a significant association between PPI use and risk of developing CDI (odds ratio [OR] = 1.26, 95% confidence interval (CI), 1.12–1.39) as compared with non-users.

Even when compared to other gastric acid-suppressive medication, a recent meta-analysis showed that the use of PPI increased the risk of hospital-acquired CDI (OR = 1.386, 95% CI 1.152–1.668) when compared to H2-antagonist [92].

Given that PPIs are overprescribed in surgical settings, consideration should be given to stop PPIs, when they are not necessary, especially in patients at high risk of CDI.

#### Nasogastric tube

The risk of poor clinical outcomes of CDI in patients with nasogastric tube (NGT) insertion is still controversial. In order to assess the outcomes of CDI in patients with NGT insertion, a systematic review and meta-analysis was recently published [93].

Eight observational studies were included in the analysis to assess the association between NGT insertion and risk of poor outcome of CDI. The pooled relative risk (RR) of severe or complicated clinical outcomes of CDI in patients with NGT insertion was 1.81 (95% CI 1.17–2.81).

This study demonstrated a statistically significant association between NGT insertion and risk of poor outcomes of CDI. This finding may impact clinical management and primary prevention of CDI. Avoidance of unnecessary NGT uses would improve the clinical outcomes of CDI.

#### Surgery

Reports have linked the development of CDI in surgical patients to the widespread use of broad-spectrum antibiotics, and the increasing number of elderly and immunocompromised patients undergoing surgical interventions [17, 94, 95].

Abdelsattar et al. [18] prospectively identified postoperative patients with laboratory-confirmed CDI following general, vascular, or gynecological surgeries at 52 academic and community hospitals in the state of Michigan, USA between July 2012 and September 2013. The highest rates of CDI occurred after lower-extremity amputation (2.6%), followed by bowel resection or repair (0.9%) and gastric or esophageal surgeries (0.7%). Gynecological and endocrine surgeries had the lowest rates of CDI (0.1% and 0%, respectively). Multivariate analysis identified increasing age, chronic immunosuppression, hypoalbuminemia (≤ 3.5 g/dL), and preoperative sepsis to be associated with postoperative CDI.

Zerey et al. [15] performed a 5-year retrospective analysis of the Agency for Healthcare Research and Quality’s National Inpatient Sample Database representing a stratified 20% sample of hospitals in the United States, from 1999 to 2003. Emergency surgery was at higher risk of CDI than elective surgery. Colectomy, small-bowel resection, and gastric resection were associated with the highest risk of CDI. Patients undergoing cholecystectomy and appendectomy had the lowest risk.

In 2010, Rodriguez et al. [96] published a retrospective analysis of all general surgery in patients admitted to a large tertiary referral general surgical unit in the UK, between March 2005 and May 2007. Multivariate analysis identified malignancy, gastrointestinal disease, anemia, respiratory disease, cardiovascular disease, diabetes mellitus, gastrointestinal surgery, and age as independently associated with *C*. *difficile*.

To assess risk factors for CDI on a surgical ward, in 2012 Kim et al. conducted a retrospective chart review of all patients admitted between January 2010 and July 2011 [97]. The rate of CDI was 0.4% (19/4720 patients). Multivariate analysis showed that colectomy and hospital stays > 10 days were the main risk factors for CDI in the surgical ward.

Using the Japanese Diagnosis Procedure Combination inpatient database, Yasunaga et al. [98] analyzed factors associated with CDI incidence and outcomes following digestive tract surgery. Of 143,652 patients undergoing digestive tract surgery, CDI was identified in 409 (0.28%) patients. High mortality, long hospital stay, and high costs were associated with post-surgical CDI.

Colorectal surgery is a documented risk factor for CDI [99, 100]. Damle et al. [101] published a retrospective analysis of patients who developed CDI following colorectal resection. The authors identified adult patients undergoing colorectal surgery between 2008 and 2012 from the US University Health System Consortium database. A total of 84,648 patients met study inclusion criteria. CDI occurred in 1266 (1.5%) patients. The strongest predictors of CDI were emergency procedure, inflammatory bowel disease, and severity of illness score. CDI was associated with a higher rate of complications, intensive care unit (ICU) admission, longer preoperative inpatient stay, 30-day readmission rate, and death within 30 days compared to non-CDI patients.

Recently, a retrospective colectomy database review of the 2015 American College of Surgeons National Surgical Quality Improvement Project [102] demonstrated that stoma reversal (OR = 2.701, 95% CI 1.966–3.711; *p* < 0.001), smoking (OR = 1.520, 95% CI 1.063–2.174; *p* = 0.022), steroids (OR = 1.677, 95% CI 1.005–2.779; *p* = 0.048), and disseminated cancer (OR = 2.312, 95% CI 1.437–3.719; *p* = 0.001) were associated with CDI in the 30-day postoperative period.

In 2008, Lumpkins et al. published a retrospective observational study on the incidence of CDI in the critically injured trauma population [103]. Five hundred eighty-one consecutive critically injured trauma patients were followed prospectively for development of CDI, diagnosed by toxin assay. Among 581 patients, 19 cases of CDI were diagnosed (3.3%). ICU length of stay, duration of mechanical ventilation, and hospital length of stay were associated with CDI. The diagnosis was documented with a median delay of 17 days after admission. Fourteen patients (74%) had received antibiotics for confirmed or suspected infection prior to CDI; 4 patients (21%) received only intraoperative prophylaxis, and 1 patient had no antibiotic exposure.

#### Obesity and bariatric surgery

Obesity as a risk factor for CDI has been debated. Several reports have recently proposed obesity as a novel risk factor for CDI [104–106]. On the other hand, Punni et al. [107], in a case-control study, showed that obesity is not a risk factor for CDI. Importantly, body mass > 35 index has been shown to be an independent risk factor for CDI [108].

To investigate the impact of the two most common bariatric surgeries on CDI, Roux-en-Y gastric bypass (RYGB), and vertical sleeve gastrectomy (VSG), a retrospective cohort study was recently published [109]. CDI rates were higher after RYGB than VSG in the first 30 days (OR = 2.10; 95% CI, 1.05–4.20) with a similar but not significant trend within 31–120 days.

Knowledge about the link between obesity, bariatric surgery, and CDI is still evolving. Further studies are needed to reveal the exact mechanisms underlying this association. At this stage, we suggest high suspicion for CDI when managing patients with obesity and undergoing bariatric surgery.

#### Inflammatory bowel disease

Patients with inflammatory bowel disease (IBD) retain an increased risk of developing CDI, along with worse outcomes, higher rates of colectomy, and higher rates of recurrence [110–115].

Patients with IBD also appear to have higher rates of asymptomatic carriage of *C*. *difficile* [116]. These patients commonly receive various types of immunosuppressive drugs including steroids which have been found to increase the risk of CDI. In addition, they have a different microbiota compared to healthy subjects [117, 118].

A recent retrospective study evaluated the impact of CDI on in-hospital outcomes among adults with IBD hospitalized in the USA [119]. Using the 2007–2013 Nationwide Inpatient Sample, hospitalizations among US adults with Crohn’s disease (CD), ulcerative colitis (UC), and CDI were identified using ICD-9 coding. Hospital charges, hospital length of stay (LOS), and in-hospital mortality was stratified by CD and UC and compared. Predictors of hospital charges, LOS, and in-hospital mortality were evaluated with multivariate regression models and were adjusted for age, sex, race/ethnicity, year, insurance status, hospital characteristics, and CDI. Among 224,500 IBD hospitalizations (174,629 CD and 49,871 UC), overall prevalence of CDI was 1.22% in CD and 3.41% in UC. On multivariate linear regression, CDI was associated with longer LOS among CD (coefficient: 5.30, 95% CI 4.61–5.99; *p* < 0.001) and UC (coefficient 4.08, 95% CI 3.54–4.62; *p* < 0.001). Higher hospital charges associated with CDI were seen among CD (coefficient $35,720, 95% CI $30,041–$41,399; *p* < 0.001) and UC (coefficient $26,009, 95% CI $20,970–$31,046; *p* < 0.001). CDI among IBD was associated with almost threefold greater risk of in-hospital mortality.

The clinical presentation of an IBD exacerbation and CDI often is indistinguishable and requires a high index of suspicion for adequate treatment [6]. As the symptoms of CDI and an exacerbation of IBD (diarrhea, abdominal pain, fever, and leukocytosis) overlap, the diagnosis of CDI may be delayed [120]. In addition, in IBD patients with ileostomies, the development of acute enteritis manifested as an increase in ileostomy output, nausea, fever, and leukocytosis may also indicate CDI. The same is true for pouchitis, which presents as an increase in the number of stools per day [121]. In one study, 10.7% of patients with ileal pouch anal anastomosis, presenting with pouchitis, were found to have CDI [122].

Due to high rates of asymptomatic colonization by *C*. *difficile* in patients with IBD, only patients with increased diarrhea or new symptoms potentially due to CDI should be tested for *C*. *difficile* toxin. Typical findings of CDI on colonoscopy are often absent in patients with IBD (0–13% of cases) [123] which may be attributed to a weakened inflammatory response. There is no evidence that one antibiotic regimen is better than another for the treatment of CDI in IBD patients. In a survey of North American gastroenterologists, there was no agreement on combination of antibiotics and immunomodulators in patients with an IBD flare and CDI [124]. The American College of Gastroenterology recommended with low-quality supporting evidence, that ongoing immunosuppression can be maintained in patients with CDI and that escalation of immunosuppression should be avoided [125].

An expert review to synthesize the existing evidence on the management of CDI in patients with underlying inflammatory bowel disease was published in 2017. The review suggested six simple advices of best practice [126].

Physicians should remain alert to the possibility of CDI in a patient with an IBD exacerbation to ensure rapid diagnosis and treatment. Early surgical consultation is also key for improving outcomes of patients with severe disease. Colectomy with preservation of the rectum may need to be considered for severely ill IBD patients with CDI.

#### Immunocompromised patients

The rate of CDI is increased in solid organ transplant recipients due to ongoing immunosuppression and antibiotic use [127].

It has also been reported that cancer patients have a higher risk compared with non-cancer patients [128] due to chemotherapy causing immunosuppression [129, 130].

Patients with HIV/AIDS are also at high risks of being infected with *C*. *difficile* too. The risk is stronger in those with low absolute CD4 T cell counts or those who meet clinical criteria for AIDS [131].

The increased risk may be partially attributed to frequent hospitalization, exposure to antibiotics, and antibiotic prophylaxis for opportunistic infections, but HIV-related alterations in fecal microbiota, gut mucosal integrity, and humoral and cell-mediated immunity may also likely play a role [132].

## Risk factors for community-acquired *C*. *difficile* infection

Although predominantly associated with the inpatient health care population, CDI originating in the community has been increasingly reported. The predominant *C*. *difficile* ribotypes isolated in the hospital setting correspond with those isolated in the community, suggesting that transmission between these two settings is occurring [133].

In 2011, an estimated 159,000 community-associated CDI (CA-CDI) occurred in the USA, representing 35% of the total CDI burden [134].

Risk factors may include increasing outpatient antibiotic prescriptions, acid-suppression medications, asymptomatic carriers in the community, and food or water contamination [135]. A sub-group analysis of a population-based epidemiological study of CDI in Olmsted County, Minnesota in 1991–2005 [136], identified 157 CA-CDI cases (75% women), with a median age of 50 years. Among them, 40% required hospitalization, 20% had severe, and 4.4% severe-complicated infection, while 20% had treatment failure and 28% had recurrent CDI.

A case-control study from ten US sites from October 2014 to March 2015 analyzed risk factors for CA-CDI [137]. Case patients were defined as persons aged ≥ 18 years with a positive *C*. *difficile* specimen collected as an outpatient or within 3 days of hospitalization who had no admission to a health care facility in the prior 12 weeks and no prior CDI diagnosis. Each case patient was matched to one control (persons without CDI). Participants were interviewed about relevant exposures; multivariate conditional logistic regression was performed. More case patients than controls had prior outpatient health care (82.1% vs. 57.9%; *p* < 0.0001) and antibiotic (62.2% vs. 10.3%; *p* < 0.0001) exposures. In multivariate analysis, antibiotic exposure—that is, cephalosporin (adjusted matched odds ratio [AmOR], 19.02; 95% CI 1.13–321.39), clindamycin (AmOR, 35.31; 95% CI 4.01–311.14), fluoroquinolone (AmOR, 30.71; 95% CI 2.77–340.05), and beta-lactam and/or beta-lactamase inhibitor combination (AmOR, 9.87; 95% CI 2.76–340.05)—emergency department visit (AmOR, 17.37; 95% CI 1.99–151.22), white race (AmOR 7.67; 95% CI 2.34–25.20), cardiac disease (AmOR, 4.87; 95% CI 1.20–19.80), chronic kidney disease (AmOR, 12.12; 95% CI 1.24–118.89), and IBD (AmOR, 5.13; 95% CI 1.27–20.79) were associated with CA-CDI.

A systematic review and meta-analysis investigated the association between medications and comorbidities with CA-CDI [138]. Twelve publications (*n* = 56,776 patients) met inclusion criteria. Antimicrobial (OR = 6.18, 95% CI 3.80–10.04) and corticosteroid (OR = 1.81, 95% CI 1.15–2.84) exposure were associated with increased risk of CA-CDI. Among the comorbidities, IBD (OR = 3.72, 95% CI 1.52–9.12), renal failure (OR = 2.64; 95% CI 1.23–5.68), hematologic malignancy (OR = 1.75; 95% CI 1.02–5.68), and diabetes mellitus (OR = 1.15; 95% CI 1.05–1.27) were associated with CA-CDI. Antimicrobial exposure was associated with a higher risk of CA-CDI in the USA, whereas PPI exposure was associated with a higher risk in Europe. The risk of CA-CDI associated with antimicrobial exposure greatly increased in adults older than 65 years.

## Risk factors for recurrent CDI

Recurrent CDI (RCDI) can be defined as reappearance of symptoms within eight weeks following the completion of a course of therapy with complete resolution of symptoms.

The key to preventing recurrent infection is identifying those patients at the greatest risk [139].

In a meta-analysis, Garey et al. [140] found that continued use of non-*C*. *difficile* antibiotics after diagnosis of CDI (OR = 4.23; 95% CI 2.10–8.55; *p* < 0.001), concomitant receipt of antacid medications (OR = 2.15; 95% CI 1.13–4.08; *p* = 0.019), and older age (OR = 1.62; 95% CI 1.11–2.36; *p* = 0.0012) were associated with increased risk of recurrent CDI. Other factors identified in individual studies include age, hospital exposure, comorbid conditions, severe underlying illness, hypoalbuminemia, impaired humoral immunity, poor quality of life, disease severity, and previous recurrent CDI [141–144].

In order to evaluate current evidence of risk factors for recurrent CDI, a systematic review and meta-analysis [145] analyzed 33 studies (18,530 patients). The most frequent independent risk factors for recurrent CDI were age ≥ 65 years (RR = 1.63, 95% CI 1.24–2.14; *p* = 0.0005), additional antibiotics during follow-up (RR = 1.76; 95% CI 1.52–2.05; *p* < 0.001), use of PPIs (RR = 1.58; 95% CI 1.13–2.21; *p* = 0.008), and renal failure (RR = 1.59; 95% CI 1.14–2.23; *p* = 0.007). The risk was also increased in patients previously on fluoroquinolones (RR = 1.42; 95% CI 1.28–1.57; *p* < 0.001).

## Clinical manifestations

The spectrum of symptomatic CDI ranges from mild diarrhea to severe disease or fulminant colitis and as many as 30% of patients may develop recurrent CDI [146, 147].

Though diarrhea is the hallmark symptom of CDI, it may not be present initially, possibly due to colonic dysmotility either from previous underlying conditions or possibly from the disease process itself [148].

This is especially important in surgical patients who may have a concomitant ileus. Therefore, in surgical patients, it is important to have a high index of suspicion for the development of CDI.

### Mild-moderate CDI

Diarrhea may be accompanied by mild abdominal pain and cramps and if prolonged may result in altered electrolyte balance and dehydration. When this occurs in patients with severe comorbidity, particularly after surgery, non-severe CDI may increase morbidity significantly [149].

### Severe CDI

Severe CDI is associated with increased abdominal cramping and pain as well as systemic features such as fever, leukocytosis, and hypoalbuminemia. The absence of diarrhea may signal a progression to fulminant infection [150]. Though a wide variety of severity predictors for severe CDI has been described [151–156], international consensus for the definition of severe CDI is lacking [6, 7].

A systematic review identifying risk factors for adverse outcomes of CDI was published by Abou Chakra et al. in 2012 [154]. Except for leukocytosis, albumin, and age, there was much heterogeneity in the data and most studies were limited by small sample sizes.

To investigate the prognostic value of fever, leukocytosis, and renal failure, in 2012 Bauer et al. [153] analyzed the database of two randomized controlled trials, which contained information on 1105 patients with CDI. They found that both leucocytosis and renal failure were useful predictors of in severe CDI. Miller et al. [155] in 2013 subsequently published an analysis of the same two clinical therapeutic trials to validate a categorization system to stratify CDI patients into severe or mild-moderate groups. A combination of five simple and commonly available clinical and laboratory variables (ATLAS) measured at the time of CDI diagnosis were able to accurately predict treatment response to CDI therapy. The ATLAS criteria included age, treatment with systemic antibiotics, leucocyte count, serum albumin, and serum creatinine levels.

Any of the following may be predictors of severe CDI:

- WBC > 15 × 10^9^/L
- Rise in serum creatinine level (≥ 133 μM/L or ≥ 1.5 times premorbid level)
- Temperature > 38.5 °C
- Albumin < 2.5 g/dL

It has been recently demonstrated that human serum albumin is capable to bind *C*. *difficile* toxin A and B thus impairing their internalization into host cells; this could partially explain the increased CDI severity experienced by hypoalbuminemic patients [157].

The progression to fulminant *C*. *difficile* colitis is relatively infrequent [158] (1–3% of all CDI) though mortality in this group of patients remains high due to the development of toxic megacolon with colonic perforation, peritonitis, and septic shock and subsequent organ dysfunction. Systemic symptoms may not merely result from toxin-induced inflammatory mediators released locally in the colon but likely to the toxins spread into the bloodstream [36, 159, 160].

Studies have demonstrated a significant rise in the number of cases of fulminant colitis associated with multiple organ failure and increased mortality in recent years associated with the hypervirulent 027 strain of *C*. *difficile* [161, 162]. Early diagnosis and treatment is therefore important in reducing the mortality associated with fulminant colitis. Patients who present organ failure including increased serum lactate or vasopressor requirements should be assessed immediately with regard to early operative intervention [162].

## Recurrent CDI

Recurrence of symptoms after initial therapy for *C*. *difficile* develops in 10–30% of cases, and presents a clinical challenge [144, 163–167]. For a patient with 1–2 previous episodes, the risk of further recurrences is 40–65%.

Recurrences are associated with an impaired immune response to *C*. *difficile* toxins and/or alteration of the colonic microbiota.

RCDI may be either a consequence of germinating resident spores remaining in the colon after antibiotic treatment has stopped, or re-infection from an environmental source.

Even though consensus regarding factors associated with CDI recurrence is not universal, algorithms have been developed to predict CDI recurrence with good sensitivity [168].

Ultimately, distinction between recurrence and reinfection can only be achieved if the strain of *C*. *difficile* is “typed” using molecular epidemiology [169].

Recurrent episodes are less severe compared to initial episodes: in a Canadian study, the authors reported a decline in the proportion of severe cases according to the number of recurrent episodes (47% for initial episodes, 31% for first recurrences, 25% for second, and 17% for third) [170].

## Additional significant consequences of CDI

Patients who develop CDI have increased hospital length-of-stay, higher medical care costs, more hospital re-admissions, and higher mortality [171–173]. These consequences are also found in surgical patients with CDI.

In the Zerey et al. analysis [15], CDI was an independent predictor of increased length of stay, which increased by 16.0 days (95% CI 15.6–16.4 days; *p* < 0.0001). Total charges increased by $77,483 (95% CI $75,174, $79,793; *p* < 0.0001), and there was a 3.4-fold increase in the mortality rate (95% CI 3.02–3.77; *p* < 0.0001) compared with patients who without *C*. *difficile infection*.

In the Abdelsattar et al. study [18], postoperative CDI was independently associated with increased length of stay (mean, 13.7 days vs. 4.5 days), emergency department presentations (18.9 vs. 9.1%), and readmissions (38.9% vs. 7.2%, all *p* < 0.001).

Data from Nationwide Inpatient Sample database in 2011 of patients who underwent vascular surgery [174] showed that in patients who had experienced CDI, the median length of stay was 15 days (IQR 9, 25 days) compared to 8.3 days for matched patients without CDI, in-hospital mortality 9.1% (compared to 5.0%), and $13,471 extra cost per hospitalization. The estimated cost associated with CDI in vascular surgery in the USA was about $98 million in 2011. Similarly, data from the National Inpatient Sample in patients undergoing lumbar surgery found that CDI increased length of stay by 8 days, hospital costs by 2-fold, and increased inpatient mortality by 36-fold [175].

Higher mortality was also observed for liver transplant recipients (from 2000 to 2010) at a Detroit hospital [176].

The ACS-NSQIP database from 2005 to 2010 was used by Lee et al. to study emergently performed open colectomies for a primary diagnosis of *C*. *difficile* colitis in the USA [177]. The overall mortality was 33% (111/335).

A study was performed to quantify additional hospital stay attributable to CDI in four European countries, by analyzing nationwide hospital-episode data [5]. Patients in England had the longest additional hospital stay attributable to CDI at 16.09 days, followed by Germany at 15.47 days, Spain at 13.56 days, and The Netherlands at 12.58 days. Propensity score matching indicated a higher attributable length of stay of 32.42 days in England, 15.31 days in Spain, and 18.64 days in the Netherlands. Outputs from this study consistently demonstrate that in European countries, in patients whose hospitalization is complicated by CDI, the infection causes a statistically significant increase in hospital length of stay.

## Recommendations for the management of CDI

### Infection prevention and control

An infection control “bundle” strategy should be used to successfully control CDI outbreaks. The “bundle” approach should include multifaceted interventions including antibiotic stewardship, hand hygiene, isolation measures, and environmental disinfection.

1. Proper antibiotic stewardship in both selecting an appropriate antibiotic and optimizing its dose and duration to prevent and cure an infection may prevent the emergence of *C*. *difficile* (Recommendation 1 B).

As CDI is thought to follow disruption of normal bacterial flora of the colon, a consequence of antibiotic use [178], it is logical that antibiotic stewardship programs may be useful in preventing CDI [179]. Good antibiotic stewardship involves ensuring appropriate antibiotic choices and optimizing antibiotic doses and duration of treatment to prevent and cure an infection while minimizing toxicity and conditions conducive to the development of CDI.

In order to estimate the effectiveness and safety of interventions to improve antibiotic prescribing practices in hospital inpatients in 2017, a systematic review including 221 studies (58 RCTs, and 163 NRS) was published [180]. The results showed a very low level of evidence regarding the effect of interventions to reduce CDI (median − 48.6%, interquartile range − 80.7% to − 19.2%; seven studies).

Another systematic review and meta-analysis quantified the effect of both persuasive (education and guidance) and restrictive (approval required, removal) antimicrobial stewardship programs for CDI [179]. A significant protective role (overall RR = 0.48, 95% CI 0.38–0.62) was found, with the strongest evidence for restrictive program and those with the longest duration. Cephalosporins and quinolones reduction should be an important target for stewardship programs, with a significant expected impact on the incidence of CDI [181, 182].

2. *C*. *difficile* carriers should be placed in contact (enteric) precautions (Recommendation 1 B). Even if further studies are warranted to establish the benefit of screening and the efficacy of infection control measures for asymptomatic carriers.

Prompt identification of patients with CDI is essential, so that appropriate isolation precautions can be put into effect [183].

This is particularly important in reducing environmental contamination as spores can survive for months in the environment [184], despite regular use of environmental cleaning agents.

It is important to place patients suspected of having CDI on contact precautions before diagnostic laboratory test confirmation if there is a lag before test results are available [185].

Contact (enteric) precautions in patients with CDI should be maintained until the resolution of diarrhea, which is demonstrated by passage of formed stool for at least 48 h. There are no studies demonstrating that further extension of contact precautions results in reductions in CDI incidence.

*C*. *difficile* carriers should be placed in a private room [186] with en-suite hand washing and toilet facilities. If a private room is not available, known CDI patients may be cohorted in the same area [187] though the theoretical risk of transfection with different strains exists. This is supported by a retrospective cohort of 2859 patients published by Chang et al. [188]. Non-infected patients who were roommates or neighbors of a patient with CDI were at higher risk of nosocomial acquisition of CDI (RR 3.94; 95% CI 1.27–12.24).

Recently, there has been growing interest in asymptomatic carriage/colonization of *C*. *difficile* since asymptomatic carriers are considered a reservoir for *C*. *difficile*. Colonization by toxigenic *C*. *difficile* strain seems to be associated with increased risk of progressing to CDI. Zacharioudakis et al. [48] showed that carriers of toxigenic strains are at a higher risk for the development of an infection compared to non-colonized patients. On the other hand, patients colonized by non-toxigenic strains may be even protected from developing CDI [189]. Conversion of a non-toxigenic strain to a toxin producer by horizontal gene transfer makes the risk assessment of colonization really challenging [190]. More data are needed to assess the precise role of the microbiota and the conditions allowing progression from asymptomatic colonization to CDI, in particular the recognition of the mechanism which may trigger toxin production. Based on current data, screening for asymptomatic carriers and an eradication of *C*. *difficile* is not indicated because *C*. *difficile* colonization is not believed to be a direct independent precursor for CDI. *C*. *difficile* asymptomatic carriers may also play a role in spore dissemination in the hospital and many cases of CDI are thought to be attributable to cross-contamination from asymptomatic carriers. Curry et al. [191] examined patients for *C*. *difficile* colonization and found that 29% of CDIs were linked to asymptomatic *C*. *difficile* carriers. Asymptomatic carriers who are colonized at admission appear to contribute to sustaining *C*. *difficile* transmission in the ward by the shedding of spores to the environment. The frequency of environmental contamination depends on the *C*. *difficile* status of the patient—34% of rooms of patients with asymptomatic colonization and 49% of rooms of CDI patients were found to be contaminated with *C*. *difficile* [192]. Infection control measures for asymptomatic carriers may be effective by limiting contamination of the hospital environment and health care workers’ hands, as well as by preventing direct patient-to-patient transmission. Longtin et al. [185] reported that screening of *C*. *difficile* colonization at hospital admission and contact precautions were associated with a significant decrease in the HA-CDI incidence rate (6.9 per 10,000 patient-days in the pre-intervention period vs. 3.0 per 10,000 patient-days during the intervention period; *p* < 0.001). This study provides the most convincing evidence to date for the significant effect of isolating asymptomatic *carriers.*

3. Hand hygiene with soap and water is the cornerstone of the prevention of *C*. *difficile* infection. Hand hygiene, contact precautions, and good cleaning and disinfection of patient care equipment and the environment should be used by all health-care workers in contact with any patient with known or suspected CDI (Recommendation 1 B).

In a health-care setting, transmission of *C*. *difficile* spores occurs primarily via the contaminated hands of health-care workers, but contact with a contaminated environment, contaminated utensils or medical devices has also been implicated. Hand hygiene with soap and water and the use of contact precautions along with good cleaning and disinfection of the environment and patient equipment should be used by all health-care workers in contact with any patient with known or suspected CDI. Hand hygiene is a cornerstone of prevention of nosocomial infections, including infection due to *C*. *difficile.* Alcohol-based hand sanitizers are highly effective against non-spore-forming organisms, but they do not kill *C*. *difficile* spores or remove *C*. *difficile* from the hands [193].

Though disposable glove use during care of a patient with CDI may be effective in preventing the transmission of *C*. *difficile*, these must be removed at the point of use and the hands should then be thoroughly decontaminated with soap and water.

For environmental cleaning, disinfection with sodium hypochlorite solutions are usually recommended in patient areas where *C*. *difficile* transmission is ongoing [194].

In 2016, a cross-sectional study was conducted in a tertiary care hospital to analyze the impact of location of sinks on hand washing compliance after caring for patients with CDI. Healthcare workers’ hand washing compliance was low, and a poor access to sinks was associated with decreased hand washing compliance [195].

Environmental decontamination of clinical areas, ideally using hypochlorite agents or a sporicidal product, is recommended; however, in practice, compliance with cleaning protocols is often suboptimal.

In 2017, a qualitative systematic review including 46 studies investigated the impact of specific interventions on CDI rates in acute-care hospitals. The most effective interventions, resulting in a 45% to 85% reduction in CDI, included daily to twice daily disinfection of high-touch surfaces (including bed rails) and terminal cleaning of patient rooms with chlorine-based products. Chlorhexidine bathing and intensified hand-hygiene practices were not effective for reducing CDI rates [196].

Newer alternatives for environmental decontamination have been introduced, notably hydrogen peroxide vapor (HPV) and, more recently, UV decontamination [197].

In a study conducted by McCord et al., breakpoint time series analysis indicated a significant reduction (*p* < 0.001) in the CDI rate at the time when HPV disinfection was implemented, resulting in a reduction in the CDI rate from 1.0 to 0.4 cases per 1000 patient-days in the 24 months before HPV usage compared with the first 24 months of HPV usage [198].

Recently, a systematic literature review and meta-analysis on the impact of no-touch disinfection methods to decrease HAIs was performed [199]. Statistically significant reduction in CDI (RR = 0.64; 95% CI 0.49–0.84) was observed using UV light no-touch disinfection technology. Important to point out that the new no-touch methods for room disinfection supplement, but do not replace, daily cleaning [200].

The European Society of Clinical Microbiology and Infectious Diseases (ESCMID) study group for *C*. *difficile* (ESGCD) recently published a set of guidelines regarding measures for prevention of *C*. *difficile* infection in acute healthcare settings [201]. According to the committee, it is recommended:

- To use personal protective equipment (gloves and gowns/disposable aprons) to decrease transmission of *C*. *difficile* or incidence of CDI
- To use contact precautions to decrease the transmission of *C*. *difficile* and reduce the incidence of CDI
- To introduce daily environmental sporicidal disinfection and terminal disinfection of rooms of patients with CDI to decrease the transmission of CDI
- To perform surveillance of CDI in combination with timely feedback of infection rates on both the hospital and ward level
- To implement restriction protocols of antibiotic agents/classes (effective in reducing CDI rates)
- To implement protocols to reduce the duration of antibiotic therapy (effective in reducing CDI rates)
- Educate healthcare workers on prevention of CDI to enhance their knowledge and skills on prevention strategies

It is not recommended:

- To screen for *C*. *difficile* to identify colonized/carrier patients as a way of altering the risk of developing CDI in either colonized subjects or other patients and thus reducing CDI rates
- To screen health care workers for *C*. *difficile* gut colonization as a routine control measure for CDI

### Diagnosis

4. The diagnosis of CDI should be based on clinical signs and symptoms in combination with laboratory tests. Stool testing should only be performed on diarrheal stools from at-risk patients with clinically significant diarrhea (≥ 3 loose stools in 24 h) with no obvious alternative explanation (Recommendation 1 C).
5. For patients with ileus who may be unable to produce stool specimens, polymerase chain reaction testing of perirectal swabs provides an acceptable alternative to stool specimen analysis (Recommendation 2B).

Typing is useful to differentiate *C*. *difficile* strains and to obtain epidemiological information. Different typing methods for *C*. *difficile* currently available are: restriction endonuclease analysis (REA), pulsed-field gel electrophoresis (PFGE), multi-locus sequence typing (MLST), repetitive-element PCR typing, toxin-typing, multi-locus variable-number tandem-repeat analysis (MLVA), and PCR-ribotyping [201]. *C*. *difficile* strains with increased virulence traits (hypervirulent) have been described in the last 15 years. In particular, PCR-ribotype 027, also known as North American pulsed-field gel electrophoresis type 1 (NAP1) or restriction endonuclease analysis group BI, has been associated with increased disease severity, recurrence, and significant mortality [202].

The diagnosis of *C*. *difficile* infection should be suspected in patients with acute diarrhea (≥ 3 loose stools in 24 h) with no obvious alternative explanation (such as laxative use), particularly in the setting of relevant risk factors (including recent antibiotic use, hospitalization, and advanced age).

Prompt and precise diagnosis is important for the effective management of CDI. An accurate diagnosis of CDI requires both clinical symptoms and a positive laboratory test.

Early identification of CDI allows early treatment and can potentially improve outcomes. Rapid isolation of infected patients is important in controlling the transmission of *C*. *difficile* [203]*.*

The diagnosis of CDI is based on the presence of a clinical picture compatible with CDI and microbiological evidence of free toxin and/or the demonstration of toxigenic *C*. *difficile* in a diarrhea stool sample [203]. Clinical features include diarrhea (defined as by passage of three or more unformed stools in 24 h), abdominal pain and cramps, abdominal distension, ileus (signs of severely disturbed bowel function), and toxic megacolon.

Since *C*. *difficile* can colonize the intestinal tract of healthy individuals, diagnostic testing for CDI should be performed only on diarrheic stools from symptomatic patients. Testing of formed stool can result in false positive tests, which may result in unnecessary antibiotic therapy.

One limitation of the reliance on stool specimens involves patients with suspected severe CDI complicated by ileus as those patients may be unable to produce specimens for testing. For those patients, testing of perirectal swabs may be an accurate and efficient method to detect toxigenic *C*. *difficile*. In 2012, Kundrapu et al. [204] described the results of a prospective study of 139 patients being tested for *C*. *difficile* infection by polymerase chain reaction. The sensitivity, specificity, positive predictive value, and negative predictive value of testing perirectal swabs were 95.7%, 100%, 100%, and 99.1%, respectively. The authors concluded that for selected patients, perirectal swabs provided an acceptable alternative to stool specimen analysis.

Clinical context such as a history of recent antibiotic administration and/or residence in hospital are useful in selecting patients for testing. Other signs such as fever, abdominal pain, leukocytosis, in combination with other laboratory tests (e.g., creatinine and serum lactate) are useful for defining the severity of infection.

6. Nucleic acid amplification tests (NAAT) for *C*. *difficile* toxin genes appear to be sensitive and specific and may be used as a standard diagnostic test for CDI. NAAT as single-step algorithm can increase detection of asymptomatic colonization, therefore it should be performed in patients with high suspicion for CDI or included in two-step algorithm starting with toxin-EIA (Recommendation 1 B).
7. Glutamate dehydrogenase (GDH) screening tests for *C*. *difficile* are sensitive but do not differentiate between toxigenic and non-toxigenic strains. They may be used in association with toxin A/B enzyme immunoassays (EIA) testing. Algorithms including screening with an EIA for GDH followed by a toxin assay may be suggested (Recommendation 1 B).
8. EIA for toxin A/B is fast and inexpensive and has high specificity but it is not recommended alone due to its relatively low sensitivity (Recommendation 1 B).
9. *C*. *difficile* culture is relatively slow but sensitive. It is rarely performed today as a routine diagnostic test. *C*. *difficile* culture is recommended for subsequent epidemiological typing and characterization of strains (Recommendation 1 C).
10. Repeat testing after a first negative sample during the same diarrheal episode may be useful only in selected cases with ongoing clinical suspicion during an epidemic situation or in cases with high clinical suspicion during endemic situations (Recommendation 1 C).

The best standard laboratory test for diagnosis of CDI has not been clearly established [205].

Currently, there is no single stool test that can be relied upon as the reference standard for the diagnosis of CDI. Several methods are suggested for the diagnosis of CDI, including toxinogenic culture (TC), cell cytotoxicity neutralization assay (CCNA), enzyme immunoassays (EIA) for toxins A, B, and/or glutamate dehydrogenase (GDH), and nucleic acid amplification tests (NAATs).

In the past, TC was accepted by many microbiologists as the method of choice for diagnosis of CDI. The procedure includes stool culture for *C*. *difficile* on a selective differential medium (cycloserine, cefoxitin, fructose agar, or CCFA) and an assay to test the colonies for the ability to produce toxins. Despite TC is considered the gold standard method, there are significant issues with TC including slow turnaround time and its inability to detect the presence of toxins in stool. This may also lead to false positive results as up to 7% of asymptomatic hospitalized patients may be colonized with toxigenic *C*. *difficile* [206].

*C*. *difficile* culture is also necessary for subsequent epidemiological typing and characterization of strains.

The EIA for toxin A/B has been adopted by most clinical laboratories because it is fast, convenient, and inexpensive [207]. However, studies have shown that sensitivity can be low. Toxin A + B EIA tests have a described sensitivity of 32–98% and a specificity of 84–100% [208].

GDH is an enzyme produced by *C*. *difficile* in relatively large amounts compared with toxins A and B [209, 210]. A positive GDH assay only documents the presence of *C*. *difficile* but it does not discriminate between toxigenic and non-toxigenic strains (about 20% of the *C*. *difficile* population). Therefore, a second test for toxin production is necessary for confirmation. GDH screening tests for *C*. *difficile* used in association to toxin A + B EIA testing gives an accurate test result quickly [207, 208] even if the sensitivity of such strategy is lower than NAATs.

The use of NAATs for the detection of *C*. *difficile* from diarrheal stool specimens was documented in the early 1990s. NAATs possess a series of advantages such as excellent sensitivity and specificity, low complexity, simplified reporting, reduced need for repeat testing, and improved turnaround time [209–212].

In particular, some NAATs such as multiplex NAATs can simultaneously detect *C*. *difficile* strains and toxin encoding genes from stool samples [213].

There are several commercially available NAATs, including a real-time PCR (RT-PCR) assay and loop-mediated isothermal amplification (LAMP) assay, both of which have an overall high analytical sensitivity (80–100%) and specificity (87–99%).

However, although NAATs have a high sensitivity and specificity, not all laboratories routinely perform this assay [214]. Moreover, some limitations have been associated with NAATs [215].

Although NAAT methods are considered superior to other methods of diagnosing CDI, this testing strategy is unable to accurately distinguish between *C*. *difficile* colonization and active disease, which may result in both over diagnosis and overtreatment of CDI, delaying recognition of other causes of diarrheal illness/outbreaks, and resulting in unnecessary exposure to antibiotics used to treat CDI.

A current topic of debate is whether a stool sample that was positive by a molecular assay needs to be tested with a confirmatory toxin assay [216] given it can also identify toxigenic *C*. *difficile* in asymptomatic patients. This underscores the importance of only testing patients with symptoms. There is no evidence suggesting that surgical patients should be diagnosed any differently than general medical patients [217]. It has already been highlighted that immunocompromised patients including those on glucocorticoids, or chemotherapy and post-transplant patients are at increased risk for CDI.

The issue of if or when to retest for CDI is inherently linked to the accuracy of the employed routine testing method. Methods with suboptimal sensitivity for *C*. *difficile* (e.g., stand-alone toxin EIAs) led to frequent retesting in some settings. In the absence of clear changes to the clinical presentation of suspected CDI (i.e., change in character of diarrhea or new supporting clinical evidence), repeating testing should not be performed.

11. CT imaging is suggested for patients with clinical manifestations of severe-complicated *C*. *difficile* colitis; however, its sensitivity is not satisfactory for screening purposes (Recommendation 2 B).

In certain clinical settings, adjunct testing methods such as radiologic diagnostic imaging may be useful for diagnosing CDI. Diagnostic computed tomography (CT) imaging can assist with an early diagnosis and may help determine the severity of the disease in patients with CDI [218].

CT has been studied as an imaging modality for diagnosing *C*. *difficile* colitis [219–222]. Typical CT findings of CDC include colonic wall thickening, dilation, peri-colonic stranding, “accordion sign” (high-attenuation oral contrast in the colonic lumen alternating with low-attenuation inflamed mucosa), “double-halo sign, target sign” (intravenous contrast displaying varying degrees of attenuation caused by submucosal inflammation and hyperemia), and ascites [223]. However, the most common finding, colonic wall thickening, is non-specific and can be found in other forms of colitis, although it may be more pronounced with CDI.

In the study by Kirkpatrick et al. [224], CT diagnosis of CDC had a sensitivity of 52%, a specificity of 93%, and positive and negative predictive valued 88%, and 67% respectively. Sensitivity would have been increased to 70% with no change in specificity if colonic wall thickness of greater than 4 mm had been used as a diagnostic criteria, in conjunction with the presence of the following factors, colon wall nodularity, accordion sign, peri-colonic stranding, or otherwise unexplained ascites.

12. Ultrasound may be useful in critically ill patients suspected to have pseudomembranous colitis who cannot be transported to the CT scan suite (Recommendation 2 C).

Point-of-care ultrasound may be useful in diagnosing and managing critically ill patients who cannot be moved to the radiology department [225].

Ultrasound findings of pseudomembranous colitis in severe cases include a thickened colonic wall with heterogeneous echogeneity as well as narrowing of the colonic lumen [226]. Pseudomembranes can also be visualized as hyperechoic lines covering the mucosa [226–229].

In the early stages of pseudomembranous colitis, the texture of the colonic wall is preserved. The hypoechoic edematous mucosa and muscularis propria may be thickened with the echogenic submucosa sandwiched between them. The presence of submucosal gaps may indicate extension of tissue damage into deeper structures. Intraperitoneal free fluid is seen in more than 70% of cases [224–227].

13. Flexible sigmoidoscopy may be helpful in the diagnosis of *C*. *difficile* colitis when there is a high level of clinical suspicion for *C*. *difficile* infection (Recommendation 2 B).

Endoscopy should be used sparingly to confirm the diagnosis of CDI since the diagnosis can be usually made by laboratory tests, clinical findings, and imaging. However, colonoscopy may be hazardous in the setting of fulminant colitis where there may be increased risk of perforation [169].

A study by Johal et al. [230] described the use of flexible sigmoidoscopy as a tool for the diagnosis of *C*. *difficile* colitis when stool assays were negative suggesting that sigmoidoscopy should be considered in all hospitalized patients with diarrhea in whom the stool tests for *C*. *difficile* cytotoxin and enteric pathogens are negative.

### Antibiotic therapy

14. Unnecessary antibiotic agent(s) should be discontinued if CDI is suspected (Recommendation 1 B).
15. Unnecessary PPIs should always be discontinued in patients at high risk for CDI (Recommendation 1 C).
16. Empirical therapy for CDI should be avoided unless there is a strong suspicion for CDI. If a patient has a strong suspicion for severe CDI, empirical therapy for CDI should be considered while awaiting test results (Recommendation 1 C).

In cases of suspected severe CDI, antibiotic agent(s) should be discontinued, if possible [231].

A meta-analysis addressing factors associated with prolonged symptoms and severe disease due to *C*. *difficile* showed that continued use of antibiotics for infections other than CDI is significantly associated with an increased risk of CDI recurrence [232].

If continued antibiotic therapy is required for treatment of the primary infection, antimicrobial therapy with agents that are less frequently implicated with antibiotic-associated CDI should be used; these include parenteral aminoglycosides, sulfonamides, macrolides, vancomycin, or tetracycline/tigecycline.

Although there is a clinical association between PPI use and CDI [89], no RCTs studies have studied the relationship between discontinuing or avoiding PPI use and risk of CDI. Thus, a strong recommendation to discontinue PPIs in patients at high risk for CDI regardless of need for PPI will require further evidences. However, stewardship activities to discontinue unneeded PPIs are strongly warranted.

Antibiotic therapy is the first choice for CDI, and specific antibiotic therapy guideline recommendations should be based on the severity of the disease.

When antibiotic therapy is indicated for symptomatic cases with a positive *C*. *difficile* toxin result, options include metronidazole, oral or intraluminal vancomycin, and oral fidaxomicin [233–239].

17. Oral metronidazole should be limited to the treatment of an initial episode of mild-moderate CDI (Recommendation 2A). Oral vancomycin is recommended for treatment of patients with mild-moderate disease who do not respond to metronidazole (Recommendation 1 A). Repeated or prolonged courses of metronidazole should be avoided due to risk of cumulative and potentially irreversible neurotoxicity (Recommendation 1 B).

Although metronidazole may be associated with more frequent side effects, and there has been a significant increase in treatment failures (especially in patients infected with the emergent 027/BI/NAP1 strain), oral metronidazole 500 mg three times per day for 10 days has been used for treating mild-to-moderate cases of CDI [240]. Repeated or prolonged courses of metronidazole should be avoided due to risk of cumulative and potentially irreversible neurotoxicity [241].

In recent IDSA guidelines, metronidazole is suggested only for patients with an initial episode of non-severe CDI in settings where access to vancomycin or fidaxomicin is limited [242].

In 2015, a systematic review and meta-analysis comparing the efficacy and safety of metronidazole monotherapy with vancomycin monotherapy and combination therapy in CDI patients was published [243]. No statistically significant difference in the rate of clinical cure was found between metronidazole and vancomycin for mild CDI (OR = 0.67, 95% CI 0.45–1.00; *p* = 0.05) or between either monotherapy and combination therapy for CDI (OR = 1.07, 95% CI 0.58–1.96; *p* = 0.83); however, the rate of clinical cure was lower for metronidazole than for vancomycin for severe CDI (OR = 0.46, 95% CI 0.26–0.80; *p* = 0.006). No significant difference in the rate of CDI recurrence was found between metronidazole and vancomycin for mild CDI (OR = 0.99, 95% CI 0.40–2.45; *p* = 0.98) or severe CDI (OR = 0.98, 95% CI (0.63, 1.53); *p* = 0.94) or between either monotherapy or combination therapy for CDI (OR = 0.91, 95% CI (0.66, 1.26); *p* = 0.56). In addition, there was no difference in the rate of adverse events (AEs) between metronidazole and vancomycin (OR = 1.18, 95% CI 0.80–1.74; *p* = 0.41). In contrast, the rate of adverse effects was significantly lower for either monotherapy than for combination therapy (OR = 0.30, 95% CI 0.17–0.51; *p* < 0.0001).

However, recent data have suggested an overall superiority of vancomycin to metronidazole for the treatment of patients with CDI and oral vancomycin 125 mg four times per day for 10 days is recommended as first choice antibiotic also for moderate cases.

In 2017, in an update of a previously published Cochrane review, moderate quality evidence suggested that vancomycin is superior to metronidazole in all cases of CDI [244]. The differences in effectiveness between these antibiotics were not too large and the advantage of metronidazole is its far lower cost even if liquid vancomycin is cheaper and reduces the cost.

18. Both oral vancomycin or fidaxomicin are recommended for treatment of all patients with severe CDI (Recommendation 1 A).
19. In patients in whom oral antibiotics cannot reach the colon, vancomycin may be administered as retention enema via a large rectal tube or catheter (Recommendation 1 B).
20. Fidaxomicin may be used to treat CDI, especially in patients at higher risk for recurrence (e.g., elderly patients or those receiving concomitant antibiotics) (Recommendation 1A).

Vancomycin orally 125 mg four times daily for 10 days is considered superior to metronidazole in severe *C*. *difficile* disease [245–247]. This may reflect the superior pharmacokinetic properties of vancomycin which is concentrated in the gut lumen. Doses of up to 500 mg have been used in some patients with severe or fulminant, as defined as hypotension or shock, ileus or megacolon, CDI [7], although there is little evidence for this in the literature.

Unlike vancomycin delivered enterally, intravenous vancomycin has no effect on CDI since the antibiotic is not excreted into the colon. Vancomycin enema may be an effective therapy for patients who cannot tolerate the oral preparation or patients with ileus who have delayed passage of oral antibiotics from the stomach to the colon [248].

Trans-stoma vancomycin may also be effective in surgical patients with Hartmann resection, ileostomy, or colon diversion. A single-hospital, retrospective chart review on 47 consecutive patients with *C*. *difficile* colitis treated with intracolonic vancomycin (ICV) was published by Kim et al. in 2013 [249]. Thirty-three of 47 patients (70%) with severe *C*. *difficile* colitis responded to adjunct intracolonic vancomycin with complete resolution without surgery. Multivariate analysis suggested that failures to intracolonic vancomycin enemas occurred in patients who were older and frail with albumin < 2.5 g/dl. Early surgery should be considered for those patients. Early surgery should also be offered to those patients who are failing maximal medical therapy including ICV enemas.

Fidaxomicin orally 200 mg twice daily for 10 days may be a valid alternative to vancomycin in patients with CDI [250, 251]. Fidaxomicin was non-inferior to vancomycin for initial cure of CDI in two prospective trials [235, 236]. In a first double-blind, randomized, non-inferiority trial [237], 629 adults with acute symptoms of *C*. *difficile* infection and a positive result on a stool toxin test were enrolled and randomly assigned to receive fidaxomicin (200 mg twice daily) or vancomycin (125 mg four times daily) orally for 10 days. The rates of clinical cure with fidaxomicin were non-inferior to those with vancomycin in both the modified intention-to-treat analysis (88.2% with fidaxomicin and 85.8% with vancomycin) and the per-protocol analysis (92.1% and 89.8%, respectively). Significantly fewer patients in the fidaxomicin group than in the vancomycin group had a recurrence of the infection, in both the modified intention-to-treat analysis and the per-protocol analysis. In a second multicenter, double-blind, randomized, non-inferiority trial [238], 535 patients, 16 years or older with acute, toxin-positive CDI were randomly allocated (1:1) to receive oral fidaxomicin (200 mg every 12 h) or oral vancomycin (125 mg every 6 h) for 10 days. Non-inferiority was shown for both the modified intention-to-treat analysis (15.4% vs. 25.3%; *p* = 0.005) and the per-protocol analysis (13.3% vs. 24.0%; *p* = 0.004). Patients receiving concomitant antibiotics for other infections had a higher cure rate with fidaxomicin (46 [90.2%] of 51) than with vancomycin (33 [73.3%] of 45; *p* = 0.031).

A randomized, controlled, open-label, superiority study, recruited hospitalized adults aged 60 years and older with confirmed CDI at 86 European hospitals extended-pulsed fidaxomicin demonstrated to be superior to standard-dose vancomycin for sustained cure of CDI [252]. Between Nov 6, 2014, and May 5, 2016, 364 patients were enrolled and randomly assigned to receive extended pulsed fidaxomicin or vancomycin. Then, 362 patients received at least one dose of study medication (181 in each group). Further, 124 (70%) of 177 patients in the modified full analysis set receiving extended-pulsed fidaxomicin achieved sustained clinical cure 30 days after end of treatment, compared with 106 (59%) of 179 patients receiving vancomycin (difference 11% [95% CI, 1.0–20.7]; *p* = 0.030; OR 1.62 [95% CI, 1.04–2.54]). Incidence of treatment-emergent adverse events did not differ between extended-pulsed fidaxomicin (121 [67%] of 181) and vancomycin (128 [71%] of 181) treatment arms.

Fidaxomicin may be useful for treating patients who are considered at high risk for recurrence (elderly patients with multiple comorbidities who are receiving concomitant antibiotics). However, it is important to note that no data on the efficacy of fidaxomicin in severe life-threatening disease are available.

The use of other antibiotics such as tigecycline [253, 254], fusidic acid, teicoplanin, rifamixin [238], and nitazoxanide [255] has been described in the literature, but they are not currently recommended for general use.

### Surgical management

Patients with fulminant colitis (FC) who progress to systemic toxicity require surgical intervention.

To determine clinical predictors for the development of fulminant colitis in patients with CDI, a 10-year retrospective review of FC patients who underwent colectomy was performed and compared with randomly selected age- and sex-matched non-fulminant CDI patients at a single institution study by Girotra et al. in 2012 [256]. Predictive clinical and laboratory features included age (> 70 years), prior CDI, profound leukocytosis (> 18,000/mm^3^), hemodynamic instability, use of anti-peristaltic medications, and a clinical trial of increasing abdominal pain, distension and diarrhea.

Another important clinical feature that should be taken into account in patients who are going to experience fulminant colitis is the occurrence of a change in mental status that could reflect significant toxemia [257].

21. Patients with severe CDI who progress to systemic toxicity should undergo early surgical consultation and should be evaluated for potential surgical intervention (Recommendation 1 C).

Patients with severe CDI who progress to systemic toxicity are likely to have serious comorbidities. Delaying surgery in this group leads to increased likelihood of adverse outcomes [258], although some reports show that a short period of medical optimization can improve outcomes before colectomy [259].

There are no reliable clinical and/or laboratory findings that can predict those patients who will respond to medical therapy and those who will need surgery [260].

Data comparing mortality rates between surgical and medical treatment for fulminant *C*. *difficile* colitis were published in a systematic review by Stewart et al. [261]. Five hundred ten patients with fulminant colitis were identified in 6 studies. Emergency colectomy for patients with FC provided a survival advantage compared with continuing antibiotics. When all 6 studies numbering 510 patients were analyzed, the pooled adjusted odds ratio of mortality comparing surgery with medical therapy, and weighted by the contribution of each study, was 0.70 (0.49–0.99) leading the authors to conclude that emergency colectomy has a therapeutic role in treating complicated CDI.

Patients presenting with organ failure (acute renal failure, mental status changes, or cardiopulmonary compromise) also need prompt intervention since the timing of surgical intervention is the key for survival of patients with FC [262–265].

Seder et al. [266] described 6841 patients with CDI and showed a decreased mortality associated with surgery performed before the need for vasopressor requirement, especially in the patients < 65 years old. Hall et al. [264] reviewed 3237 consecutive cases of CDI and showed an increased mortality rate when surgical exploration was performed after intubation or the development of respiratory failure and the use of vasopressors.

Recently, a risk scoring system (RSS) for daily clinical practice was designed by van der Wilden et al. [267]. Age greater than 70 years was assigned 2 points, white blood cell counts equal to or greater than > 20,000/μL or equal to or less than 2000/μL was assigned 1 point, cardiorespiratory failure was assigned 7 points, and diffuse abdominal tenderness on physical examination was assigned 6 points. A value of 6 points was determined to be the threshold for reliably dividing low-risk (< 6) from high-risk (≥ 6) patients. Only patients with cardiorespiratory failure or diffuse abdominal tenderness were high risk.

Ferrada et al. [268] reviewed the existing literature on the treatment of CDI and published practice management guidelines (PMG) for the Eastern Association for the Surgery of Trauma (EAST). The authors strongly recommended that adult patients with CDI undergo early surgery before developing shock and requiring vasopressors. Although optimal timing remains controversial, the authors found that it was between 3 and 5 days after diagnosis in patients who are worsening or not clinically improving [268].

Many factors have been described as predictors of mortality in patients who undergo emergency surgery.

Sailhamer et al. [269] reviewed the records of 4796 inpatients diagnosed with *C*. *difficile* colitis. In 199 patients (4.1%) with fulminant CDI, the in-hospital mortality rate was 34.7%. Independent predictors of mortality included age 70 years or older, severe leukocytosis or leukopenia (white blood cell count, > or = 35,000/μL or < 4000/μL) or bandemia (neutrophil bands, > or = 10%), and cardiorespiratory failure (intubation or vasopressors). Survival rates were higher in patients who were cared for by surgical vs. nonsurgical departments.

The ACS-NSQIP database from 2005 to 2010 was used by Lee et al. to study emergency open colectomies performed for *C*. *difficile* colitis in the USA [177]. The overall mortality was 33% (111/335). Age 80 years or older, preoperative dialysis dependence, chronic obstructive pulmonary disease, and wound class III were associated high patient mortality. Thrombocytopenia (platelet count < 150 × 10^3^/mm^3^), coagulopathy (international normalized ratio > 2.0), and renal insufficiency (blood urea nitrogen > 40 mg/dL) were also associated with a higher mortality.

A systematic review and meta-analysis of outcomes following emergency surgery for CDI was published by Banghu et al. [270]. Thirty-one studies were included, which presented data for 1433 patients. The authors concluded that the strongest predictors for postoperative death were those relating to preoperative physiological status: preoperative intubation, acute renal failure, multiple organ failure and shock requiring vasopressors.

22. Early diagnosis and treatment is important to reduce the mortality associated with fulminant colitis.
23. Resection of the entire colon should be considered to treat patients with fulminant colitis (Recommendation 1 B). However, diverting loop ileostomy with colonic lavage is a useful alternative to resection of the entire colon (Recommendation 1 B).
24. Patients with fulminant colitis should be treated with high dose vancomycin (500 mg, 6 hourly), oral and/or by enema, in combination with intravenous metronidazole (500 mg, 8 hourly) (Recommendation 1 C).

In the Bhangu et al. meta-analysis [270], the most commonly performed operation for treatment of fulminant colitis (FC) was total colectomy with end ileostomy (89%, 1247/1401). When total colectomy with end ileostomy was not performed, reoperation to resect further bowel was needed in 15.9% (20/126). In the recent meta-analysis by Ferrada et al. [268], 17 studies comparing colectomy versus other procedures or no surgery as treatment for CDI were analyzed. The authors recommended that total colectomy (versus partial colectomy or other surgery) is the procedure of choice for patients with *C*. *difficile* colitis.

To evaluate the role of emergency colectomy in patients with FC, and to identify subgroups of patients that may benefit from it, Lamontagne et al. [271] published a retrospective observational cohort study of 165 cases of FC requiring ICU admission or prolongation of ICU stay in 2 tertiary care hospitals in Quebec, Canada. Eighty-seven (53%) patients died within 30 days of ICU admission, of whom almost half (38 of 87, 44%) died within 48 h of ICU admission. The independent predictors of 30-day mortality were leukocytosis ≥ 50 × 10^9^/L, lactate ≥ 5 mmol/L, age ≥ 75 years, immunosuppression, and shock requiring vasopressors. Patients who underwent an emergency colectomy were less likely to die than those treated medically. Colectomy was more beneficial in patients aged 65 years or more, in immunocompetent patients and in patients with a leukocytosis ≥ 20 × 10^9^/L or lactate between 2.2 and 4.9 mmol/L.

Diverting loop ileostomy with antegrade colonic lavage may be a colon-preserving alternative to total colectomy [272, 273]. A prospective, nonrandomized, historical control group study was performed at the University of Pittsburgh Medical Center and the Veterans’ Administration Healthcare System, in Pittsburgh between June 2009 and January 2011 [272]. Forty-two patients with FC were managed by a loop ileostomy, intraoperative colonic lavage with warmed polyethylene glycol 3350/electrolyte solution via the ileostomy, and postoperative antegrade instillation of vancomycin flushes via the ileostomy. There was no significant difference in age, sex, pharmacologic immunosuppression, and Acute Physiology and Chronic Health Evaluation-II scores between the studied cohort and historical controls. The operation was accomplished laparoscopically in 35 patients (83%). This treatment strategy resulted in reduced mortality compared to their historical controls. Preservation of the colon was achieved in 39 of 42 patients (93%). Of note, vancomycin antegrade enemas were continued via the ileostomy every 6 h for 10 days and this likely augmented the effect of the defunctioning surgery.

A retrospective multicenter study conducted under the sponsorship of the Eastern Association for the Surgery of Trauma to compare loop ileostomy versus total colectomy as surgical treatment for CDI was published in 2017 [274]. Data from ten centers of patients who presented with CDI requiring surgery between July 1, 2010 and July 30, 2014 were collected. When comparing colectomy and loop ileostomy, there was no statistical difference between these two operative strategies. Univariate pre-procedure predictors of mortality were age, lactate, timing of operation, vasopressor use, and acute renal failure. There was no statistical difference between the APACHE score of patients undergoing either procedure (TC, 22 vs. LI, 16). Adjusted mortality (controlled for pre-procedure confounders) was significantly lower in the loop ileostomy group (17.2% vs. 39.7%; *p* = 0.002).

### Supportive care

25. Early detection of shock and aggressive management of underlying organ dysfunction are essential for improved outcomes in patients with fulminant colitis (Recommendation 1 C). Supportive measures, including intravenous fluid resuscitation, albumin supplementation, and electrolyte replacement, should be provided to all patients with severe *C*. *difficile* infection (Recommendation 1 C).

Early detection and prompt aggressive treatment of the underlying organ dysfunction is an essential component in the management of CDI in critically ill patients.

Severe CDI may present with a fulminant course and may be associated with great morbidity and high mortality. Physiologic support including invasive monitoring in an intensive care unit and aggressive resuscitation are often necessary in fulminant colitis. Diarrhea results in significant volume depletion and electrolyte abnormalities, and fluid and electrolyte imbalance should be promptly corrected.

Although it has been debated, albumin supplementation in patients with severe hypoalbuminemia (< 2 g/dl) should be considered as a supportive measure and also to exploit its anti-toxin properties [275].

The expert panel suggests measuring intra-abdominal pressure (IAP) when any known risk factor for intra-abdominal hypertension (IAH)/abdominal compartment syndrome (ACS) is present.

### RCDI

Recurrence is diagnosed when CDI recurs < 8 weeks after the resolution of a previous episode, provided the symptoms from the previous episode resolved after completion of the initial treatment and other causes have been excluded. Symptomatic recurrent *C*. *difficile* infection (RCDI) occurs in approximately 20% of patients and is challenging [141]. Therefore, patients with recurrent CDI should therefore be treated by experienced clinicians.

26. Agents that may be used to treat the first recurrence of CDI include vancomycin (particularly if metronidazole was used for the first episode) or fidaxomicin. (Recommendation 1 B).
27. Antibiotic treatment options for patients with > 1 recurrence of CDI include oral vancomycin therapy using a tapered and pulsed regimen (Recommendation 1C).

For recurrent cases of CDI, oral vancomycin 125 mg four times per day for 14 days or oral fidaxomicin 200 mg twice a day for 10 days is recommended for first recurrence.

Metronidazole is not recommended as initial treatment of recurrent CDI as sustained response rates are lower than those with vancomycin. Furthermore, metronidazole should not be used for long-term therapy because of the potential for cumulative neurotoxicity.

Vancomycin and fidaxomicin are equally effective in resolving CDI symptoms but fidaxomicin has been shown to be associated with a lower likelihood of CDI recurrence after a first recurrence [237, 238, 276]. However, there are no prospective randomized controlled trials investigating the efficacy of fidaxomicin in patients with multiple recurrences of CDI. Vancomycin is often administered using a prolonged tapered and/or pulsed regimen which may be more effective than a standard 10 to 14 days course, although no RCTs have been reported in second or subsequent CDI recurrences [146].

### Probiotics

28. Limited direct evidence exists to support the use of probiotics in the management of a first episode of CDI as an adjunctive treatment to antibiotics for immunocompetent patients Recommendation 2 B).

The altered composition of gut microbiota in the setting of *C*. *difficile* infection has raised interest in the potential role of probiotics [163]. Their use aims to re-colonize and restore the diversity of flora following the disruption due to antibiotic treatment and *C*. *difficile* overgrowth.

There is limited direct evidence to support the use of probiotics in the primary prevention of CDI.

Data for primary prevention of CDI often arises from prevention of antibiotic-associated diarrhea trials with CDI as a secondary outcome and are often underpowered for CDI. Thus, meta-analyses may be useful to evaluate if specific probiotics are efficacious for CDI, as this statistical method utilizes the increase in power resulting from pooling different studies together. However, since the recent finding that the efficacy of probiotics are both strain-specific and disease-specific [277], for valid conclusions to be reached, the meta-analysis must assess efficacy within subgroups of identical probiotic strains (or mixture of strains) and for the same type of disease. A meta-analysis of 22 randomized controlled trials using sub-group analysis for 5 different types of probiotics for primary prevention of CDI found 4/5 (*Saccharomyces boulardii* I-745, *Lactobacillus casei* DN114001, a mixture of *Lactobacillus acidophilus* and *Bifidobacterium bifidum*, and another mixture of three Lactobacilli strains [*L*. *acidophilus* CL1285, *L*. *casei* LBC80R, *Lactobacillus rhamnosus* CLR2]) were effective and one type (*L*. *rhamnosus* GG) was not effective [278]. Other systematic reviews and meta-analyses report a protective effect of probiotics [279–284], but reviews exploring the contribution of probiotics in CDI prevention can be limited due to heterogeneity between studies, inadequate study power, or significant levels of missing outcome data. In addition, many reviews still fail to account for strain-specificity and pool different types of probiotics together in their analysis [280, 281, 284]. The short-term use of probiotics appeared to be safe and effective when used along with antibiotics in patients who are not immunocompromised or severely debilitated. Probiotics should not be administered to patients at risk of bacteremia or fungemia.

29. Prophylactic probiotics may be considered for inpatients receiving antibiotics during high-risk period (such as outbreaks) before the disease develops (Recommendation 2 C). Probiotics should be not used in immunocompromised patients (Recommendation 2 C).

Several types of probiotics have been tested on a facility-level intervention as part of an infection control bundle for CDI. In an effort to reduce hospital-wide CDI rates (especially in hospitals having CDI outbreaks), probiotics were given to newly admitted patients receiving antibiotics and continued during either the duration of the antibiotic or duration of the patient’s stay. Although lacking in the rigorous strength from randomized trials, these hospital studies showed a significant reduction of CDI rates for some types of probiotics (*L*. *casei* Shirota, *Lactobacillus plantarum* 299v, and a mixture of three lactobacilli strains, Bio-K+) [278]. This three lactobacilli strain mixture (*L*. *acidophilus* CL1285, *L*. *casei* LBC80R, and *L*. *rhamnosus* CLR2) has been tested in seven other hospitals and found to be effective in reducing CDI rates [285]. However, other types of probiotics need further research, particularly in those at high risk of CDI. Probiotics are contraindicated for immunocompromised patients due to a rare, but serious risk of bacteremia.

30. Probiotics for prevention of recurrent CDI may be an effective adjunct to standard antibiotic treatment (vancomycin) in patients with at least one prior episode of CDI (Recommendation 2 B).

There have been many case reports and case series reporting fewer recurrences of CDI when some probiotics were used as an adjunctive treatment with vancomycin or metronidazole. However, there are fewer randomized trials for this adjunctive therapy. Two randomized controlled trials found significantly fewer CDI patients developed recurrences when *Saccharomyces boulardii* I-745 was combined with standard antibiotic therapy [286, 287]. The first trial demonstrated a lower CDI recurrence rate compared with a placebo control group (26% vs. 45%, respectively) [283] and the second trial found that the combination of *S*. *boulardii* (1 g/day) with high dose vancomycin (2 g/day) was more effective than high dose vancomycin and placebo (17% vs. 50% recurrence rate) [284]. The probiotic was not able to reduce CDI recurrences when combined with a lower dose of vancomycin (500 mg/day) or with metronidazole (1 g/day). Other studies with Lactobacillus strains (*L*. *rhamnosus* GG or *L*. *plantarum* 299v) were stopped prematurely due to enrollment problems [146]. There have no published trials currently combining probiotics with fidaxomicin.

### Fecal microbiota transplantation

31. Fecal microbiota transplantation (FMT) may be an effective option for patients with multiple recurrences of CDI who have failed appropriate antibiotic treatments (Recommendation 2 C).

FMT has been considered as an alternative therapy to treat RCDI [283–293]. It involves infusing intestinal microorganisms (in a suspension of healthy donor stool) into the intestine of patients to restore the intestinal microbiota.

The rationale of FMT is that disruption of the normal balance of colonic flora allows *C*. *difficile* strains to grow and produce CDI. By reintroducing normal flora via donor feces, the imbalance may be corrected, and normal bowel function re-established [288].

FMT has not been widely adopted as a therapeutic tool probably due to concerns regarding safety and acceptability [258].

A systematic literature review of FMT treatment for RCDI and pseudomembranous colitis was published in 2011 by Gough et al. [289]. In 317 patients treated across 27 case series and reports, FMT was highly effective, showing disease resolution in 92% of cases. In those studies, 35% of patients received FMT via enema, with a response rate of 95%; 23% patients received FMT via naso-jejunal tube by gastroscope, with a response rate of 76%; and 19% via colonoscopy, with a response rate of 89%. Effectiveness varied by route of instillation, relationship to stool donor, volume of FMT given, and treatment before infusion.

Another systematic review was published by Cammarota et al. [290]. Twenty full-text case series, 15 case reports, and 1 randomized controlled study were included for the final analysis. Almost all patients treated with donors’ fecal infusion had experienced recurrent episodes of CD-associated diarrhea despite standard antibiotic treatment. Of a total of 536 patients treated, 467 (87%) had resolution of diarrhea. Diarrhea resolution rates varied according to the site of infusion: 81% in the stomach, 86% in the duodenum/jejunum, 93% in the cecum/ascending colon, and 84% in the distal colon. No severe adverse events were reported with the procedure.

Recently, a review to evaluate the efficacy of FMT in treating recurrent and refractory CDI was published [291]. Thirty-seven studies were included; 7 randomized controlled trials and 30 case series. FMT was more effective than vancomycin (RR = 0.23, 95% CI 0.07–0.80) in resolving recurrent and refractory CDI. Clinical resolution across all studies was 92% (95% CI 89–94%). A significant difference was observed between lower gastrointestinal (GI) and upper GI delivery of FMT 95% (95% CI 92–97%) vs. 88% (95% CI 82–94%) respectively (*p* = 0.02). There was no difference between fresh and frozen FMT 92% (95% CI 89–95%) vs. 93% (95% CI 87–97%) respectively (*p* = 0.84). Administering consecutive courses of FMT following failure of first FMT resulted in an incremental effect. Donor screening was consistent but variability existed in recipient preparation and volume of FMT. Serious adverse events were uncommon.

Although FMT has high success rates with long-term durability [292], few disadvantages still exist. In particular, the manipulation of feces and the classical enteral administration methods are not only laborious but tend to make the procedure rather unattractive for physicians and patients.

In the context of these disadvantages, few efforts have been made to enhance the feasibility and social acceptance of microbiota transplantation.

FMT may be administered via enemas or as a slurry given via a nasogastric tube.

One systematic review which compared various routes of administration included a total of 182 patients (148 received FMT via colonoscopy and 34 received FMT via nasogastric tube) from 12 published studies [293]. Recurrence of CDI after FMT was similar in both the colonoscopy group (8/148, 5.4%) versus the nasogastric tube group (2/34, 5.9%) (*p* = 1.000). However, the overall rate of cure after FMT was slightly higher in patients receiving FMT by colonoscopy: 85.3% (29 patients, 29/34) in the nasogastric tube group and 93.2% (138 patients, 138/148) in the colonoscopy group (*p* = 0.162).

A larger and more recent systematic review of 14 studies including 305 patients and comparing FMT delivery by upper and lower gastrointestinal routes also favored lower gastrointestinal delivery [294]. At 30 and 90 days, the risk of clinical failure was 5.6% and 17.9% in the upper gastrointestinal group compared with 4.9% and 8.5% in the lower GI delivery route group, respectively.

More recently, encapsulated preparations of FMT have been used with success. This strategy has the advantage of being less invasive and simpler, which may also result in improved cost-effectiveness [295–298].

In 2014, Youngster et al. [296] reported their experience with frozen FMT capsules in 20 patients who had RCDI. Fourteen patients (70%) had resolution of diarrhea after a single treatment, and 4 patients responded after a second treatment, with a clinical resolution rate of 90%.

Patients who are immunocompromised are at increased risk of CDI. During the last 2 years, the first data on FMT in immunocompromised patients began to appear in the medical literature [299].

A multicenter retrospective series on the use of FMT in immunocompromised patients with recurrent, refractory, or severe CDI was published in 2014 [300]. Immunosuppression included HIV/AIDS (3), solid organ transplantation (19), oncologic condition (7), immunosuppressive therapy for IBD (36), and other medical conditions/medications (15). This series demonstrated the effective use of FMT for CDI in immunocompromised patients with few serious adverse events.

With the increased awareness of the role of native gut microbiome and its role in the gut brain axis, there have been concerns about the long-term effect of transplanted stool, and how the new gut microbiome can affect brain function and immune responses.

### Monoclonal antibodies

32. Coadjuvant treatment with monoclonal antibodies (bezlotoxumab) may prevent recurrences of CDI, particularly in patients with CDI due to the 027 epidemic strain, in immunocompromised patients and in patients with severe CDI (Recommendation 1 A).

Since the expression of clostridial toxins (TcdA and TcdB) is mandatory for the development of CDI, the development of monoclonal antibodies aimed at preventing the cytotoxic effect of these toxins is a potential strategy for controlling the disease. In 2016, the FDA approved bezlotoxumab to reduce the recurrence of CDI in adult patients receiving antimicrobial therapy for CDI who are at high risk of CDI recurrence. Bezlotoxumab (MK-6072) is a human monoclonal antibody which reduces recurrent CDI by blocking the binding of *C*. *difficile* toxin B to host cells, thus limiting epithelial damage and facilitating recovery of the microbiome [301]. Besides bezlotoxumab, another human monoclonal antibody, actoxumab (MK-3415), was recently designed to neutralize *C*. *difficile* toxin.

The data from two double-blind, randomized, placebo-controlled, phase 3 trials, MODIFY I and MODIFY II, involving 2655 adults receiving oral standard-of-care antibiotics for primary or recurrent *C*. *difficile* infection showed that bezlotoxumab achieved a significant benefit over placebo in the treatment of recurrent CDI. Participants received an infusion of bezlotoxumab (10 mg/kg of body weight), actoxumab plus bezlotoxumab (10 mg/kg each), or placebo; actoxumab alone (10 mg/kg) was given in MODIFY I but discontinued after a planned interim analysis. The primary end point was recurrent infection (new episode after initial clinical cure) within 12 weeks after infusion in the modified intention-to-treat population [302].

In both trials, the rate of recurrent *C*. *difficile* infection was significantly lower with bezlotoxumab alone than with placebo (MODIFY I: 17% [67 of 386] vs. 28% [109 of 395]; adjusted difference, − 10.1 percentage points; 95% CI, − 15.9 to − 4.3; *p* < 0.001; MODIFY II: 16% [62 of 395] vs. 26% [97 of 378]; adjusted difference, − 9.9 percentage points; 95% CI, − 15.5 to − 4.3; *p* < 0.001) [303].

A post-hoc analysis of pooled monoclonal antibodies for *C*. *difficile* therapy (MODIFY) I/II data assessed bezlotoxumab efficacy in participants with risk factors for RCDI including age ≥ 65 years, history of CDI, compromised immunity, severe CDI, and ribotype 027/078/244 [304]. Although the patients with only one of the risk factors may benefit from bezlotoxumab, patients with at least three risk factors appeared to have the greatest risk reduction with bezlotoxumab.

### Intravenous immunoglobulin

33. Intravenous immunoglobulin (IVIG) should only be used as adjunct therapy in patients with multiple recurrent or fulminant CDI until results from large, randomized controlled trials are available (Recommendation 2 C).

Novel treatment modalities for management of CDI have been developed. IVIG treatment is based on evidence that the level of immune response to *C*. *difficile* colonization is the major determinant of the magnitude and duration of clinical manifestations. Passive immunization with IVIG has been successful in several small series. A review by Abourgergi et al. [305] of 15 small, mostly retrospective and non-randomized studies, documented success with IVIG in the treatment of protracted, recurrent, or severe CDI. The authors concluded that IVIG should only be used as adjunct therapy until results from large, randomized controlled trials are available. Two small retrospective matched cohort studies were published that compared the clinical efficacy of the addition of IVIG to conventional CDI treatment [306, 307]. Neither of these studies found significant differences between the compared cohorts in the main clinical outcomes, although Shahani et al. [306] noted that in their IVIG cohort, there were significantly older patients with more severe CDI than in the control group. It is reasonable to utilize IVIG therapy in patients diagnosed with hypogammaglobuminemia based on the confirmation of IgG levels below the normal laboratory range.

### Enteral nutrition in CDI

34. Tube feeding patients should be clinically assessed due to their risk for developing CDI (Recommendation 2 C).

It is widely accepted that enteral nutrition (EN) maintains gut mucosal integrity which leads to decreased intestinal permeability, decreased infections, and an improved immunological status. EN during episodes of diarrhea may be well tolerated and may improve enterocyte healing and maintenance of enzyme activity [308–310]. Enteral nutrition, however, has also been associated with increased risk of CDI [310]. Bliss et al. evaluated 76 tube-fed and non-tube-fed hospital patients for the development of CDI [311]. Patients were controlled for age, severity of illness, and duration of hospitalization. Patients who were tube-fed were statistically more likely to develop CDI (20% vs. 8% *p* = 0.03). One of the reasons may be prolonged use of elemental diets. It is known that critically ill patients tolerate feeding well if the feed is given in elemental form and delivered beyond the stomach into the jejunum because it is totally absorbed within the upper small intestine [312], depriving the colonic microbiota of their source of nutrition, such as dietary fibers, fructose oligosaccharides, and resistant starch [313]. The resultant suppression of colonic fermentation may therefore lead to the disruption of the normal gut flora and the creation of a “permissive” environment for *C*. *difficile* colonization and subsequent infection. In feeding tube patients, the conversion of elemental diet feeding to a diet containing adequate indigestible carbohydrate after the first week of critical illness may, in theory, be beneficial.

Puri et al. [314] reported that daily concomitant treatment with 4 g cholestyramine in patients receiving long-term intravenous ceftriaxone (2 to 4 g ceftriaxone daily, for an average of > 10 weeks) was associated with CDI in only 3 out of 46 patients (6.5%) compared with 23.1% of those receiving ceftriaxone alone. Cholestyramine (or colestyramine) is a hydrophilic, water insoluble, non-digestible basic anion-exchange resin which can bind luminal TcdA and TcdB.

### Anti-motility agents

35. The use of anti-peristaltic agents for the treatment of CDI should be discouraged. If anti-peristaltic agents are used to control persistent symptoms in patients with CDI, they must always be accompanied by medical therapy (Recommendation 2 C).

A review of the literature regarding anti-motility treatment of CDI found 55 patients with CDI who were exposed to anti-motility agents [315]. Nine patients (16%) died, and 27 patients (49%) had unknown outcomes. Seventeen patients (31%) with CDI developed colonic dilation; 5 of these patients with severe CDI died. However, all patients who experienced complications or died were given anti-motility agents alone initially, without an appropriate antibiotic and 23 patients who received metronidazole or vancomycin co-administered with the anti-motility agent experienced no complications. Further study of the role of anti-motility agents in providing symptomatic relief and reducing environmental contamination with infectious stool may be warranted though, until there is clear evidence of benefit, their use in patients with CDI should be avoided.

## Conclusions

In the last three decades, the worldwide increase in CDI incidence has been particularly apparent among surgical patients, becoming a global public health challenge. Therefore, prompt and precise diagnosis is paramount for the effective management of CDI, allowing both the immediate implementation of infection prevention and control strategies, and the optimization of treatment in surgical patients, considering the most recent changes introduced in the management of this infection.

## Abbreviations

CDI: *Clostridium difficile* infection
FC: Fulminant colitis
RCDI: Recurrent *Clostridium difficile* infection
